# The PTSD-Bone Axis: Evidence, Mechanisms, and Management

**DOI:** 10.1007/s11914-026-00957-2

**Published:** 2026-03-16

**Authors:** Olivia X. Jones, Kirsten D. Kelly, Stephanie K. Khoo, Ryan R. Kelly, Sara J. Sidles, Amanda C. LaRue

**Affiliations:** 1 Lowcountry Center for Veterans Research, Charleston, SC USA; 2 Research Service, Ralph H. Johnson VA Health Care System, Charleston, SC USA; 3 Department of Pathology and Laboratory Medicine, Medical University of South Carolina, Charleston, SC USA

**Keywords:** Post-traumatic stress disorder (PTSD), Bone remodeling, Musculoskeletal trauma, Osteoporosis, Neuroendocrine-immune axis, Sex-specific vulnerability

## Abstract

**Purpose of Review:**

This review explores the relationship between post-traumatic stress disorder (PTSD) and bone health, focusing on how chronic psychological stress influences skeletal integrity through neuroendocrine, immune, and behavioral pathways.

**Recent Findings:**

Clinical and preclinical studies demonstrate PTSD is associated with reduced bone mineral density, impaired healing, and fracture risk. Mechanistic insights implicate hypothalamic-pituitary-adrenal (HPA) axis dysregulation, sympathetic nervous system (SNS) overactivation, and chronic inflammation in disrupting bone remodeling. Additional risk modifiers include sex-specific biology, early-life adversity, and glucocorticoid sensitivity. However, selective serotonin reuptake inhibitors (SSRIs), serotonin-norepinephrine reuptake inhibitors (SNRIs), anti-inflammatory agents, and emerging tools like exosomal profiling and microbiome modulation show promise in mitigating stress-related bone loss.

**Summary:**

PTSD contributes to skeletal fragility through complex, multisystem mechanisms. Trauma-informed care integrating bone health screening and personalized interventions may improve psychological and musculoskeletal outcomes. Future research should prioritize longitudinal, mechanistic studies to guide holistic management of trauma-related disease.

## Introduction

Post-traumatic stress disorder (PTSD) and other mental health disorders are increasingly recognized as potential contributors to skeletal fragility. Observational studies link PTSD to increased fracture risk and impaired healing [1]. PTSD is a chronic psychiatric condition characterized by intrusive memories, hyperarousal, and dysregulated stress-response systems following trauma [2]. The skeleton is essential for structural support, mineral storage, and hematopoiesis, yet its integrity can be compromised by systemic factors beyond traditional risk determinants. Recent research has expanded our understanding of the cyclical relationship between mental health conditions and bone remodeling [3, 4]. Emerging evidence suggests psychiatric disorders, particularly PTSD, may influence bone remodeling through neuroendocrine, immune, and psychological mechanisms. Dysregulation of the hypothalamic-pituitary-adrenal (HPA) axis, chronic inflammation, and sympathetic nervous system (SNS) overactivation, hallmarks of PTSD, are implicated in impaired osteoblast function, enhanced osteoclast activity, and reduced bone mineral density (BMD) [5]. These findings indicate that PTSD and related disorders can contribute to osteoporosis, rather than simply coexist with it.

PTSD is a complex psychiatric condition that develops in some individuals following exposure to a traumatic event. Clinically, it is characterized by four primary symptom clusters: intrusion (e.g., flashbacks and nightmares), avoidance of trauma-related stimuli, negative alterations in cognition and mood, and marked alterations in arousal and reactivity [6]. PTSD is also characterized by significant systemic biochemical dysregulation. A hallmark of PTSD is the disruption of the hypothalamic-pituitary-adrenal (HPA) axis, often manifesting as altered cortisol dynamics and a diminished capacity for the body to terminate the stress response [6]. This is frequently accompanied by chronic overactivation of the SNS, leading to sustained elevations in circulating catecholamines such as norepinephrine and epinephrine. Furthermore, PTSD is increasingly recognized as a state of systemic inflammation, evidenced by elevated levels of pro-inflammatory cytokines, including interleukin-6 (IL-6), tumor necrosis factor-alpha (TNF-α), and C-reactive protein (CRP), alongside increased markers of oxidative stress. Together, these factors create a persistent, altered physiological state that affects multiple organ systems.

The goal of this review is to synthesize recent evidence on the biological and clinical intersections between PTSD and bone health. Human and preclinical findings are first summarized to link PTSD with skeletal outcomes, followed by a detailed examination of the underlying molecular mechanisms, including neuropsychiatric pathways and anti-inflammatory signaling. We then discuss key risk modifiers such as sex differences, early-life adversity (ELA), and postmenopausal osteoporosis. This is followed by an exploration of the cyclical links between PTSD and musculoskeletal trauma, as well as emerging areas such as exosome signaling and the microbiome-gut-bone-brain axis. Finally, we address clinical implications, management strategies, and research gaps to inform integrated approaches for mitigating stress-related bone deterioration.

## Clinical Evidence Linking PTSD to Skeletal Outcomes

Clinical research in populations with high PTSD prevalence indicates PTSD is associated with reduced BMD and increased fracture risk [7]. Individuals diagnosed with PTSD have been linked to poor bone healing outcomes, including higher periprosthetic fracture rates, which may reflect stress-induced bone loss [8]. Recent preclinical evidence supports these observations. Mouse models of PTSD and chronic stress demonstrate clinically relevant alterations in bone tissue microarchitecture, including significant trabecular bone loss [9]. Social isolation stress alone reduces trabecular and cortical bone parameters in male mice [10], and even studies primarily focused on skeletal muscle show that chronic stress significantly reduces BMD in mice, suggesting that stress-induced physiological changes extend to the musculoskeletal system [11].

The rationale for these outcomes is rooted in a “brain-bone axis,” where neurological and psychiatric distress directly disrupt bone homeostasis and elevate fracture risk [3]. Mechanistic insights suggest chronic psychological stress compromises skeletal integrity through hormonal imbalances, inflammation, and SNS activation [12]. Specifically, dysregulated stress-response systems, compounded by oxidative stress, chronic inflammation, and hormonal imbalances, can shift bone homeostasis toward bone loss. Consistent with this, Luo et al. discusses how cellular senescence accelerates bone degeneration through the accumulation of p16lnk4a and senescence-associated secretory phenotype (SASP) factors [13]. These stress-related factors drive a lineage shift in bone marrow mesenchymal stem cells (BMSCs), favoring adipogenesis over osteogenesis. Furthermore, the resulting pro-inflammatory environment characterized by elevated cytokines like interleukin-1 (IL-1), IL-6, and TNF-α exacerbates the imbalance in bone remodeling by promoting osteoclast activity and impairing osteogenesis. These findings emphasize the need to explore cellular and molecular mechanisms underlying stress-related skeletal deterioration, as summarized in Table 1.

**Table 1:** Recent Clinical and Preclinical Evidence Linking PTSD to Bone Health. Human and animal studies demonstrate associations between PTSD, chronic stress, and skeletal fragility. These effects may be mediated by primary pathophysiology, behavioral changes, and pharmacological interventions. Clinically supported impacts on BMD, fracture risk, and healing outcomes are noted, along with molecular and cellular mechanisms implicated in stress-induced bone remodeling. Each effect is marked with directional arrows to indicate whether the process is increased (↑) or decreased (↓)

Study Type	Population/Model	Key Findings	Ref.
Clinical (Observational)	17,474 adults aged ≥ 65 years from Fukushima Prefecture post-Great East Japan Earthquake	↑ Fracture risk; other risk factors include cancer, stroke, heart disease, diabetes, smoking, sleep dissatisfaction	[7]
	646,186 total knee arthroplasty (TKA) patients; 7,381 PTSD patients matched to 7,381 non-PTSD patients	↑ Emergency department visits, ↑ infections, ↑ periprosthetic fractures, ↑ revision rates	[8]
Clinical (Review)	General population; physiological and behavioral factors	↑ Hormonal changes, ↑ inflammation, ↑ SNS hyperactivation, ↑ behavioral risks; may influence epigenetics and socioeconomic disparities	[13]
	General population; aging individuals; postmenopausal women	↑ Prevalence (200 M); ↓ estrogen, ↑ oxidative stress, ↑ inflammation, ↑ hormonal imbalance	[14]
Translational (Review)	Various neurological disease categories (e.g., autoimmune, developmental, dementia-related, movement, neuromuscular, stroke, trauma, psychological)	↓ Bone homeostasis, ↓ bone mass, ↑ fracture risk; possible bidirectional brain–bone effects	[3]
	Individuals with psychological stress disorders (including PTSD) and osteoporosis risk	↓ BMD, ↑ fracture risk, ↓ healing outcomes; complicates treatment adherence	[4]
Preclinical (Experimental)	Adult female C57BL/6 mice exposed to inescapable foot shock and social isolation	↑ Long-term behavioral and inflammatory changes; ↓ trabecular bone; ↓ bone material quality; ↑ aberrant architecture and cellular activity	[9]
	Adult male and female C57BL/6J mice subjected to 4 weeks of social isolation (1 mouse/cage) versus grouped housing (4 mice/cage)	↓ Trabecular and cortical bone parameters in males; no bone loss in females. Males showed reduced osteoblast and osteoclast gene expression; females showed ↑ bone resorption–related gene expression without structural loss.	[10]
	Male C57BL/6 mice subjected to water-immersion restraint stress	↑ Serum corticosterone, ↓ thymus volume, ↓ BMD, ↓ body weight, ↓ muscle mass, ↓ grip strength; ↓ cross-sectional area of type 2b muscle fibers; ↑ Redd1, FoxO1, FoxO3, KLF15, Atrogin1, Fkbp51; ↓ p-S6 and p-4E-BP1.	[11]

## Mechanistic Pathways for PTSD & Skeleton Interactions

### HPA/GR Axis

Bone remodeling is a highly coordinated process that is driven by a tightly coupled balance between osteoblast-driven formation and osteoclast-mediated resorption and coordinated by osteocytes, the primary mechanosensors in bone. Remodeling can be heavily influenced by comorbid conditions like PTSD. These effects occur through multiple biological pathways (summarized in Table 2). One of the primary pathways is through glucocorticoid signaling. Glucocorticoids are regulated by the HPA axis and play a dual role in bone physiology. Under normal conditions, they support skeletal homeostasis; however, they can contribute to bone deterioration when chronically elevated [14].

**Table 2:** Mechanistic Pathways Connecting PTSD and Bone Remodeling. PTSD-related biological pathways discussed in this review are divided into four categories: endocrine, neurogenic, immune, and behavioral. Key mediators and downstream effects on bone integrity are summarized, highlighting how stress-driven signaling alters osteoblast and osteoclast activity, bone formation, and resorption. Each effect is marked with directional arrows to indicate whether the process is increased (↑) or decreased (↓)

Mechanism	Key Mediators & Pathways	Impact on Bone Health	Ref.
Endocrine	**Direct Glucocorticoid Action**: Osteoblasts, Osteocytes, and Osteoclasts, Autophagy, Angiogenesis	Decreased survival and differentiation of osteoblasts and osteocytes, increased apoptosis and ferroptosis, impaired angiogenesis, shift toward marrow adiposity	[14, 15, 17], 22– [27, 32]
	**Wnt & BMP Signaling**: Dkk1, Sost (Sclerostin), sFRP-1, Runx2, β-catenin, AKT, GSK3β	Increased Wnt and BMP antagonists, suppressed Runx2 and β-catenin signaling, reduced mineralization and bone formation	[16, 19, 21, 23, 24, 27, 32]
	**RANKL/OPG & Bone Remodeling**: TRAP activity, ROS	Shift in RANKL to OPG ratio favoring resorption, increased osteoclast formation and activity, uncoupling of bone formation and resorption	[14, 15, 17, 18, 26, 32]
	**Growth Factors**: IGF-I, IGF-II, IGFBPs (1 to 6)	Reduced IGF-I expression and receptor sensitivity, inhibited osteoblast differentiation and formation	[20]
	**HPA Axis & GR Receptor**: Epigenetics (NR3C1), Cortisol sensitivity, Lipid storage	Chronic HPA dysregulation and GR hypersensitivity via promoter methylation, increased bone marrow adipose tissue, systemic bone loss	[16, 28–32]
Neurogenic	**Sympathetic Nervous System**: Norepinephrine, Catecholamines, β-adrenoreceptors	SNS overactivation leading to norepinephrine release, inhibited bone growth and fracture healing, increased resorption via β-adrenergic signaling	[33–35]
Immune	**Inflammation & Myelopoiesis**: PTSD, Chronic Stress, HSC niche, Cytokines	Shift toward myeloid-biased hematopoiesis, increased inflammatory cytokine release, uncoupling of osteogenesis and angiogenesis	[36, 37, 40, 41]
	**Neuroimmune Suppression**: Blunted GM-CSF, Microglial activity	Blunted neuroimmune feedback leading to sustained SNS overactivity and systemic inflammation, increased osteoclast activation	[39]
	**Protective Pathways**: SCF, IL-4, TGF-β signaling	Counter-regulatory signaling that potentially protects against stress-induced bone loss by modulating osteoblast activity	[38]
	**Cellular Aging**: Senescence (SASP), DNA repair, Marrow aging	Increased marrow aging markers and SASP, reduced DNA repair capacity, accelerated aging of the bone environment, increased inflammatory signaling	[41]
Behavioral	**Circadian & Sleep**: Sleep deprivation, Circadian misalignment, Oxidative stress	Disrupted cortisol rhythms and inflammatory cytokine profiles, oxidative damage to bone cells, reduced bone formation	[42–45]
	**Psychological**: PTSD (Dysphoric arousal symptoms)	Chronic psychological stress driving sleep disturbances and metabolic impairment, increased risk of osteopenia and reduced bone density	[46]

Glucocorticoids impair osteoblast differentiation by suppressing bone morphogenetic protein-2 (BMP-2) signaling and key osteoblast transcription factors, such as runt-related transcription factor 2 (RUNX2), reducing osteocalcin (OCN) expression, and activating cellular stress responses (e.g., endoplasmic reticulum stress) [15]. These actions are mediated through the glucocorticoid receptor (GR), which plays a role in maintaining bone homeostasis under normal physiological conditions, but, when chronically activated by excess glucocorticoids, leads to reduced osteoblast function and bone formation. Notably, female GR conditional knockout (GR-CKO) mice exhibited persistent bone loss and increased marrow adiposity with age, highlighting the importance of GR signaling in bone maintenance [16]. These disruptions collectively impair osteoblast function and contribute to skeletal degeneration.

Dysregulated GR signaling impairs osteoblast differentiation and promotes osteoclastogenesis [17] by increasing receptor activator of nuclear factor kappa-B ligand (RANKL) expression and reducing osteoprotegerin (OPG) [18]. Glucocorticoids inhibit key osteogenic pathways, including insulin-like growth factor-1 (IGF-1), Wingless-related integration site (Wnt), and bone morphogenetic protein (BMP) signaling, while regulating antagonists, such as Dickkopf-1 and sclerostin [19–21]. They further shift the lineage commitment of mesenchymal stromal cells toward adipogenesis at the expense of osteoblast formation [22].

At the cellular level, glucocorticoids induce apoptosis, senescence, and ferroptosis in osteoblasts and osteocytes, diminishing vascular endothelial growth factor (VEGF) and other osteogenic signals, leading to impaired angiogenesis and reduced skeletal vasculature that compromises bone integrity and mechanosensing [23, 24]. Pharmacological doses of glucocorticoids triggered autophagy in osteocytes, which could progress to apoptosis under excessive exposure [25]. This process is mediated by caspase-3 activation and elevated reactive oxygen species (ROS), which disrupts key survival pathways: protein kinase B (AKT) and Wnt/β-catenin [26, 27]. A key mechanism underlying glucocorticoid-induced apoptosis is the disruption of the balance between pro-survival and pro-apoptotic proteins. Glucocorticoids suppress B-cell lymphoma-extra large (Bcl-XL), which normally protects osteoblasts and osteocytes from cell death, while upregulating the apoptotic mediators, Bcl-2-like protein 11 (Bim) and Bcl-2 homologous antagonist/killer (Bak) [15, 27].

Beyond skeletal effects, dysregulated GR signaling is a hallmark of PTSD, where altered HPA axis signaling drives maladaptive stress responses. While the initial trauma often triggers an acute surge in cortisol, the transition to chronic PTSD is characterized by a unique physiological shift leading to chronically reduced cortisol. Unlike chronic stress, which is characterized by elevated cortisol that can contribute to bone deterioration, individuals with PTSD often paradoxically exhibit blunted basal cortisol levels but still experience bone deterioration. This has been attributed to a longitudinal increase in negative feedback sensitivity, GR hyperresponsiveness, and chronic HPA axis dysregulation [28–31]. Under these conditions, even low basal cortisol levels can overactivate hypersensitive receptors, leading to sustained bone suppression. Emerging evidence also suggests that administration of glucocorticoids directly following trauma may reduce PTSD symptoms and risk, which could have longer-term systemic implications, including skeletal protective effects [32]. More research is needed to fully understand the interactions between PTSD, cortisol, and bone maintenance.

### Sympathetic Nervous System (SNS)

In PTSD, SNS overactivation disrupts bone homeostasis by impairing microvasculature and increasing bone resorption, which leads to skeletal fragility [5]. A mouse study demonstrated that chronic psychosocial stress impairs bone metabolism and fracture healing through neutrophil-derived catecholamines that act on β2-adrenoreceptors in chondrocytes to prevent their transdifferentiation into osteoblasts [33]. More recent evidence shows both acute and chronic psychological stress, especially when associated with hypertension, can accelerate bone resorption via immune and endocrine pathways [34]. SNS overactivation in mental health disorders, such as depression, leads to sustained norepinephrine release. This release suppresses osteoblast proliferation and promotes osteoclastogenesis via β2-adrenergic signaling, thereby favoring bone resorption over formation [35]. This sympathetic surge further alters the immune landscape by promoting the systemic release of pro-inflammatory cytokines and mobilizing neutrophils, which act as secondary mediators of bone loss. Together, these findings highlight how chronic psychological stress alters bone remodeling through neuroendocrine and sympathetic pathways, setting the stage for immune-mediated mechanisms that further exacerbate diminished skeletal integrity.

### Immune System

Bone marrow niches dynamically regulate hematopoietic stem cell (HSC) survival, quiescence, and differentiation, adapting to aging and acute stress in ways that shape systemic immune responses and stress-related pathology [36]. Katrinli et al. comprehensively reviewed how PTSD-driven SNS activation and glucocorticoid resistance drive chronic inflammation and immune dysfunction, which may impair bone remodeling and contribute to skeletal fragility [37]. Genetic evidence from Mendelian randomization suggests that higher levels of stem cell factor (SCF) and interleukin-4 (IL-4) are associated with a reduced risk of PTSD [38]. This implies that a deficiency in these protective cytokines, potentially mediated by the *POGZ* and *LRIG2* genes, may contribute to the skeletal fragility seen in this population. Although PTSD is usually linked to elevated systemic inflammation, Bonomi et al. found that the brain’s immune response can actually become suppressed [39]. Reduced microglial activation and blunted granulocyte-macrophage colony-stimulating factor (GM-CSF) signaling may impair the brain’s ability to monitor and dampen systemic stress signals. Without this neuroimmune regulation, the SNS can become chronically overactive, leading to a sustained release of norepinephrine that triggers bone resorption.

Beyond sympathetic signaling, other neuroendocrine pathways also disrupt bone marrow homeostasis. Specifically, Mosialou et al. demonstrated that stress-induced arginine vasopressin (AVP) promotes myeloid-based differentiation of HSCs [40]. This dysregulated differentiation increases the production of pro-inflammatory cells that infiltrate the brain to promote neuroinflammation, contributing to the systemic nature of this stress-induced pathology [40]. Chronic stress further accelerates biological aging in these bone marrow leukocytes by enhancing cellular senescence markers, thus impairing DNA repair, and promoting inflammatory signaling [41].

### Sleep & Circadian Rhythm

Disrupted sleep and circadian rhythm disturbances, common in PTSD and depression, have also been implicated in bone loss [42]. Sleep deprivation alters cortisol rhythms and inflammatory cytokine profiles, contributing to impaired bone formation and increased bone resorption [43]. Sleep loss induces systemic inflammation, disrupts immune regulation, and elevates pro-inflammatory cytokines, which can negatively affect bone remodeling and skeletal integrity [44]. Insomnia is a common comorbidity of PTSD that can cause oxidative damage, which may impair glucose metabolism and further exacerbate systemic stress responses [45]. Both full and subthreshold PTSD have been associated with increased occurrence of sleep disorders, with dysphoric arousal symptoms primarily driving sleep difficulties [46].

## Biological Mechanisms and Molecular Pathways Connecting PTSD to Bone Remodeling

### Neuropsychiatric & Anti-Inflammatory Interventions

Psychological stress-related disorders have well-documented clinical effects and treatment interactions [4]. Neuropsychiatric regulation can influence skeletal integrity through neuroendocrine and inflammatory pathways. For example, CE-123 is a cognitive enhancer that mitigates PTSD-induced bone loss by modulating dopamine signaling in the brain [47]. Selective serotonin reuptake inhibitors (SSRIs) are widely prescribed for individuals with depression due to their favorable safety profile, general tolerability, yet they have garnered attention for their potential impact on bone metabolism and fracture risk [48]. While antidepressants have been associated with adverse skeletal effects, emerging data suggest nuanced outcomes. Duloxetine hydrochloride, a serotonin-norepinephrine reuptake inhibitor (SNRI), demonstrated protective effects in ovariectomized mouse models by suppressing osteoclastogenesis through inhibition of RANKL-induced signaling cascades, suggesting a potential therapeutic application for preventing bone loss in postmenopausal women [49]. Similarly, fluoxetine administration in depressive rat models rescued impaired fracture healing and led to improved radiological and histological findings, indicating relevance for patients with comorbid depression and skeletal injury [50]. These studies illustrate that pharmacological modulation of neuropsychiatric pathways can influence bone health via neuroendocrine, serotonergic, and pathways, suggesting targeted approaches may preserve skeletal integrity under psychological stress.

In addition to SSRIs and SNRIs, other neuropsychiatric medications also affect bone metabolism. Antipsychotics, particularly those associated with hyperprolactinemia, have been linked to decreased BMD and increased fracture risk due to prolactin-mediated suppression of sex hormones and direct effects on dopamine and serotonin receptors in bone cells [51, 52]. Beta blockers, prescribed for anxiety and cardiovascular conditions, may reduce bone resorption by dampening sympathetic activity to offer protective skeletal benefits [53, 54]. However, outcomes vary depending on the specific agent and patient populations, and further research is warranted to clarify their role in skeletal metabolism. Opioids, prescribed for chronic pain and PTSD-related symptoms, impair bone integrity by inducing hypogonadism, reducing testosterone and estrogen levels, and suppressing bone formation, while also directly affecting bone cells via mu-opioid receptor activation [55, 56]. However, these pharmacological treatments primarily target hormonal or neurological pathways and often carry off-target skeletal risks, leaving the underlying systemic inflammation of PTSD unaddressed. This gap has spurred interest in using anti-inflammatory strategies as adjuncts to counteract the inflammatory environment and preserve bone integrity.

Pharmacological modulators like nonsteroidal anti-inflammatory drugs (NSAIDs) have complex, dose-dependent effects on bone health. For instance, low-dose aspirin enhances osteoblast activity and inhibits bone resorption via cyclooxygenase-independent pathways [57]. However, clinical studies reveal mixed outcomes, with some reporting modest increases in BMD but no significant reduction in fracture risk [58]. Emerging anti-inflammatory strategies have shown promise in mitigating the skeletal and psychological consequences of chronic stress. ARA290, a non-erythropoietic erythropoietin mimic peptide, targets the EPOR/CD131 receptor complex to promote anti-inflammatory M2 microglial polarization [59]. This mechanism has been shown to reverse depression-like behavior and reduce bone marrow immune cell activation without altering erythropoiesis [59]. Similarly, systemic administration of the beta blocker propranolol in chronically stressed rats attenuated inflammation and limited bone resorption [60]. This suggests that the SNS contributes to skeletal fragility through a dual-action feed-forward loop, where direct adrenergic signaling on bone cells is further amplified by a sympathetic-driven inflammatory environment [60]. Phyllanthin, a natural, non-nutrient plant compound with anti-inflammatory properties, also improved BMD and exhibited antiosteoporosis effects in preclinical models [61]. These findings indicate that pharmacological interventions targeting neuropsychiatric and inflammatory pathways may offer a multifaceted approach to maintaining bone health under chronic stress conditions.

## Risk Modifiers

### Neurobiological and Skeletal Implications for Women with PTSD

Emerging evidence suggests PTSD impacts bone integrity through sex-specific biological mechanisms, with females demonstrating a unique molecular vulnerability. Women are disproportionately affected by PTSD and often exhibit more robust inflammatory responses, particularly IL-6, IL-1β, TNF-α, and CRP, which can accelerate bone resorption [62, 63]. This vulnerability is heavily modulated by estrogen, which normally regulates the HPA axis and protects bone. Consequently, the decline of estrogen during menopause removes this protective buffer, making postmenopausal women particularly susceptible to stress-induced osteoporosis [64]. Given this clinical evidence, menopause hormone therapy (MHT) has been explored as a strategy to counteract this vulnerability, demonstrating protective effects on bone mineral density in postmenopausal women [65].

This concept of “estrogen buffering” is supported by preclinical data where social isolation led to significant trabecular and cortical bone loss in adult male mice, whereas female mice exhibited increased expression of bone-resorption related genes without immediate structural deterioration [10]. However, the isolated females still exhibited a high-turnover phenotype with increased markers of bone formation and resorption. This suggests that, while females possess the molecular priming of bone decay, the presence of estrogen may maintain a compensatory balance that delays physical deterioration [10]. Furthermore, neuroimaging studies reveal that females with PTSD exhibit distinct neurobiological profiles, such as disrupted amygdala-prefrontal connectivity, which may further sensitize the HPA axis to chronic stress [66]. Together, these findings highlight a differential biological response where females may demonstrate early molecular signs of bone vulnerability that manifest as a consequence of diminished hormonal protection.

### Neurobiological and Skeletal Implications for Men with PTSD

Although PTSD is more prevalent in women, men face unique physiological contexts that may also compromise bone health. A key concern is hypogonadism, or testosterone deficiency, which is increasingly observed in men with chronic stress-related conditions, including PTSD [67]. This condition stems from disruptions in the hypothalamic-pituitary-gonadal axis, where PTSD can suppress gonadotropin release, ultimately lowering testosterone levels [68]. Testosterone plays a central role in maintaining BMD by promoting osteoblast activity and suppressing bone resorption [69]. Consequently, men with hypogonadism are at increased risk for osteoporosis and related fractures [70]. Encouragingly, it is believed that testosterone replacement therapy (TRT) can contribute to maintaining and increasing BMD among hypogonadal men [71]. Emerging evidence also suggests TRT may modulate stress responses and enhance overall functioning in men with PTSD.

### Early-Life Adversity (ELA)

Building on the molecular insights into stress-induced bone remodeling, ELA offers a developmental lens through which sex-specific vulnerability to bone loss can be understood. A study examining ELA and chronic adult stress provided evidence that stress-induced bone deterioration is highly sex-dependent. Female mice were more susceptible to ELA, whereas male mice showed greater vulnerability to stressors encountered in adulthood [72]. Notably, immunoregulatory interventions, such as the administration of *Mycobacterium vaccae*, a nonpathogenic soil bacterium, have been shown to mitigate these negative effects in both sexes [73]. This highlights a potential immunoregulatory approach for managing stress-related bone disorders.

While ELA is a well-established risk factor for developing PTSD following trauma exposure and may function as a developmental PTSD “primer”, it also independently induces microbiome-mediated bone loss that resembles stress-related pathology in female pups [74]. This early developmental window appears particularly critical in females, where ELA-induced perturbations have shown impaired bone growth and mineralization at various postnatal stages, with lasting effects on tibial length, bone microarchitecture, and gene expression related to immune and cellular homeostasis functions [75]. These studies do not suggest that ELA is the sole driver of sex differences in osteoporosis; rather, they indicate that early stress can prime the female skeleton, reducing its resilience to subsequent physiological challenges, such as adult trauma or the estrogen deficiency of menopause.

### Postmenopausal Osteoporosis

Emerging evidence indicates that the menopause transition represents a critical period of vulnerability in women with PTSD, as hormonal fluctuations exacerbate both psychological and skeletal decline. PTSD symptom severity varies across reproductive stages, with perimenopausal women exhibiting significantly higher levels of hyperarousal and depressive symptoms compared with pre- and postmenopausal groups, suggesting that fluctuating estrogen levels may intensify stress reactivity and mood dysregulation [76]. In postmenopausal women, declining estrogen further amplifies glucocorticoid sensitivity, inflammatory signaling, and SNS activity, which are all mechanisms that collectively accelerate bone resorption and increase osteoporosis risk. Midlife female Veterans with PTSD also report heightened vasomotor, urinary, and sexual symptoms, particularly linked to dysphoric arousal, highlighting overlapping pathways that influence both menopausal and PTSD-related physiology [77]. Large cohort data further support a hormonal basis for this association, as early menarche, multiple pregnancies, and menopause all increase PTSD risk, whereas lifestyle factors, such as diet and exercise, do not [78]. Despite this growing recognition, targeted psychoeducational or behavioral interventions addressing menopause and PTSD comorbidity remain limited [79]. Although direct studies examining the combined impact of PTSD, menopause, and bone loss are lacking, the mechanistic overlap suggests that hormonal decline and stress-related neuroendocrine dysregulation may synergistically exacerbate postmenopausal bone fragility.

## Cyclical Links between PTSD and Musculoskeletal Trauma

**Fig. 1:**
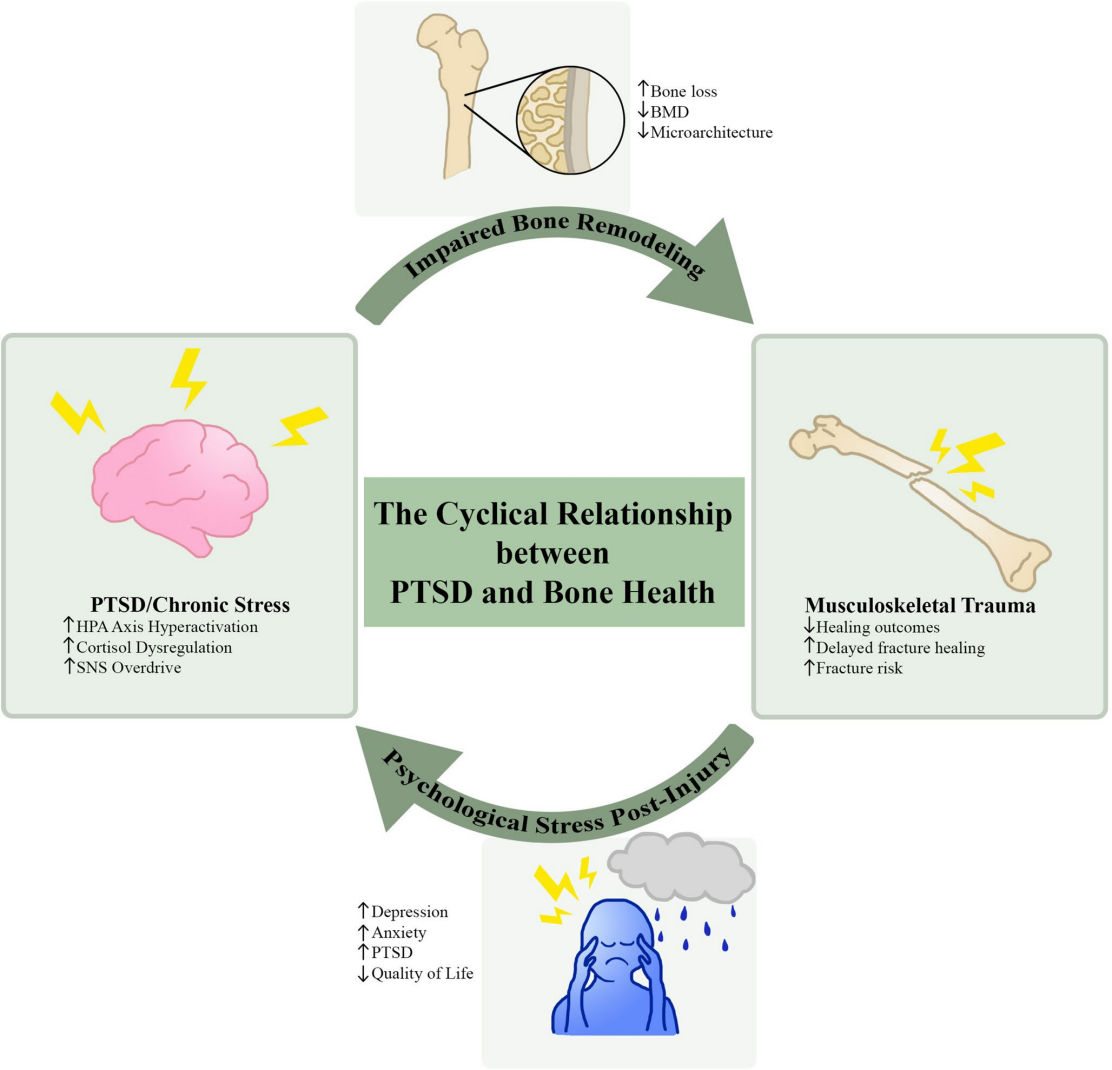
The cyclical relationship between PTSD and bone health. This schematic illustrates the bidirectional interplay between psychological stress disorders and skeletal health. PTSD and chronic stress can impair bone remodeling through HPA axis hyperactivation, cortisol dysregulation, and sympathetic nervous system (SNS) overdrive, leading to bone loss and reduced BMD. Conversely, musculoskeletal trauma and poor surgical outcomes increase psychological vulnerability, perpetuating depression, anxiety, and PTSD symptoms

While there is ample evidence that psychological stress-related disorders negatively affect bone health, musculoskeletal trauma may also exacerbate psychological distress to form a bidirectional relationship (Fig. 1). Chronic pain serves as a primary mediator in this cycle, acting as a persistent biological stressor that fuels the brain-bone axis. Although psychological stress disorders can impair bone healing, the persistent pain and poor surgical outcomes resulting from musculoskeletal trauma (such as periprosthetic factors) can perpetuate psychological vulnerability [80].

Musculoskeletal trauma leads to significant psychological consequences, including depression, anxiety, PTSD, and substance use disorders, which may, in turn, compromise fracture healing and worsen skeletal recovery [81]. Notably, patients who experienced lower limb trauma exhibited a significantly higher incidence of PTSD [82, 83]. This is particularly pertinent to populations like active military personnel, who face a heightened risk of traumatic injuries and may be more susceptible to developing PTSD [84]. Evidence suggests that the anatomical location of bone injury can influence these psychological responses, highlighting the importance of site-specific considerations into clinical care [85]. Furthermore, the specific type of trauma plays a critical role. Patients who experience fractures, amputations, or polytrauma (common in military injuries) are significantly more susceptible to PTSD, especially when recovery is complicated by poor postoperative outcomes or lasting disability [86]. This bidirectional relationship suggests traumatic bone injuries not only increase the risk of developing PTSD but that the resulting psychological distress further impairs healing, creating a cycle of worsened skeletal and mental health outcomes.

## Emerging Directions

### Exosomes

Growing evidence suggests that psychological stress alters bone physiology through systemic and cellular pathways, with extracellular vesicles (EVs), particularly exosomes, mediating this crosstalk. Exosomes are nanoscale vesicles secreted by most cell types that carry bioactive cargo, including proteins, lipids, and microRNAs (miRNAs). These vesicles act as a primary transport mechanism for the various pro-inflammatory and neuroendocrine factors identified throughout this review.

For instance, patients who experienced severe trauma exhibited altered exosomal miRNA profiles that led to suppressed bone marrow function [87]. This mechanism is further illustrated in studies of military operational stress, where intense physical and psychological strain induced significant elevations in circulating IL-6 and TNF-α alongside distinct changes in the EV profile [88]. These stress-induced changes in EVs were positively correlated with the inflammatory response, while hormones associated with anabolic, or growth-promoting, milieu significantly decreased [88].

In service members and military veterans with mild traumatic brain injury (mTBI), EV proteins and miRNAs in peripheral blood correlated with PTSD symptom severity. These altered miRNA patterns are directly associated with the previously described inflammation, neurodegeneration, and GR signaling [89]. This suggests that dysregulated miRNAs in PTSD patients modulate the inflammatory and stress-response pathways that influence bone remodeling through HPA axis dysregulation [90]. As an example, plasma exosomal miR-30a-5p has been identified as a key inhibitor of osteogenic differentiation in a depression-induced rat model [91]. Ultimately, stress-induced exosomal signaling represents a cellular mechanism that intersects with systemic pathways, including the microbiome-gut-bone-brain axis, to influence bone and mental health.

### Microbiome-Gut-Bone-Brain Axis

Emerging evidence suggests the microbiome-gut-brain axis influences both mental health and skeletal integrity. Genetic evidence from Mendelian randomization analyses indicate a potential causal relationship between gut microbiome composition and the development of PTSD [92]. These findings, which identify specific bacterial traits as drivers of trauma-related psychopathology, suggest that a dysfunctional gut-brain axis is a primary contributor to PTSD, rather than a mere consequence of stress. Individuals with PTSD often exhibit reduced microbial diversity and altered taxa, which correlate with symptom severity [93]. Accordingly, gut dysbiosis may amplify stress responses and heighten susceptibility to PTSD development.

The gut microbiome also influences neuroimmune and neuroendocrine pathways that regulate inflammation, cortisol, and neurotransmitter signaling that perpetuate stress-related pathology [94]. Beyond mental health, gut microbiota regulates bone metabolism through short-chain fatty acids, immune signals, and hormones [95]. Dysbiosis is associated with osteoporosis and disrupted osteoblast-osteoclast balance. Microbiome composition further impacts skeletal integrity and fracture risk, as certain taxa are negatively correlated with bone density and microarchitecture [96]. Chronic PTSD-related dysbiosis may exacerbate systemic inflammation and cortisol dysregulation, impair calcium absorption, and hinder bone remodeling, thereby elevating osteoporosis risk [97]. These interconnected pathways underscore the need to explore the microbiome/gut/bone/brain axis as a therapeutic target. Lifestyle interventions that restore gut health could simultaneously enhance mental resilience and skeletal outcomes.

## Clinical Implications/Management

In the context of bone health, individuals with PTSD may require tailored preventive strategies, including earlier screening with dual-energy X-ray absorptiometry (DXA), routine monitoring of vitamin D levels, and testosterone screening for symptomatic men. Beyond screening, management should incorporate lifestyle-based interventions that target the neuroendocrine-immune axis. Emerging evidence supports adopting a Mediterranean-style diet that is rich in fruits, vegetables, whole grains, and lean proteins to reduce systemic inflammation and support BMD in this population [98]. Physical activity complements these dietary benefits, with regular weight-bearing physical activities, such as resistance training or yoga, shown to preserve bone mass while improving mood and mitigating sleep disturbances common in individuals with PTSD [99–102]. These non-pharmacological approaches address core PTSD symptoms and enhance skeletal integrity through neuroimmune regulation.

To further mitigate skeletal risk, clinicians should minimize the use of sedating medications to reduce fall risk and carefully document all steroid exposure. Wearables (e.g., sleep trackers, heart rate variability, electrodermal activity, and emerging biomechanical or ultrasound sensors) can support PTSD management and bone risk surveillance by providing objective measures of adherence and symptomology [103–106]. These tools may enable personalized monitoring and timely adjustments to therapy.

It is important to note that PTSD can significantly impair an individual’s ability to adhere to medical treatments and attend scheduled healthcare appointments, which poses a major challenge to effective clinical management. Individuals with PTSD are nearly three times more likely to be nonadherent to medications than those without the disorder, with over 40% patients reporting forgetting or skipping doses [107, 108]. This high rate of nonadherence persists even after adjusting for depression and other comorbidities.

Military veterans with PTSD exhibit high dropout rates from trauma-focused therapies, often due to misunderstandings about treatment, poor therapeutic outcomes, and unaddressed emotional needs [109]. Missed appointments are also prevalent, with mental health patients missing up to 20% of scheduled visits; nearly double the rate seen in other specialties [110]. To address these barriers, trauma-informed care approaches [111], family support interventions [112], and technology-assisted adherence platforms, such as digital reminders and telemedicine [113], could be utilized to enhance engagement and continuity of care. A proactive strategy involves screening for PTSD-related barriers early, fostering trust and collaboration, and integrating flexible solutions to support sustained adherence and treatment participation.

## Limitations/Research Gaps

Despite evidence linking PTSD to compromised bone health, significant limitations and research gaps remain. Most studies are observational and struggle to separate causality from correlation because confounding factors like medication use (e.g., SSRIs), lifestyle behaviors (smoking, alcohol use, physical inactivity), and comorbid conditions (e.g., depression or chronic pain) are rarely fully controlled [5, 114]. Racial and ethnic disparities further complicate interpretation. Minority populations often experience higher PTSD prevalence and systemic barriers to care, yet few studies stratify bone health outcomes by race or examine the role of social determinants and discrimination-related stress [115, 116]. Additionally, most research focuses on older adults or postmenopausal women, leaving gaps in our understanding of men, younger populations, and those with intersecting vulnerabilities like traumatic injury or low socioeconomic status [12, 117]. Future studies should employ longitudinal designs and explore precision approaches that integrate genetic, hormonal, and psychosocial factors to clarify causality and inform targeted interventions.

## Conclusions

There is a complex interplay between PTSD and skeletal health, mediated by neuroendocrine, immune, and psychological mechanisms that disrupt bone remodeling. PTSD results in chronic HPA axis dysregulation, SNS overactivation, and persistent inflammation that collectively suppress osteoblast function and enhance osteoclast activity, predisposing individuals to osteoporosis and increasing fracture risk. Additional factors, such as glucocorticoid sensitivity, inflammatory signaling, ELA, and sex-specific biology, further amplify this vulnerability. Current pharmacological, anti-inflammatory, and lifestyle interventions show promise in mitigating stress-related bone loss. Emerging tools like exosomal profiling, microbiome modulation, and wearable sensors offer new opportunities for personalized care. Trauma-informed management that addresses the bidirectional relationship between PTSD and musculoskeletal trauma may be critical for improving both psychological and skeletal outcomes. Future research should prioritize longitudinal, mechanistic studies and integrate proactive bone screening into PTSD care to help prevent stress-induced osteoporosis and advance holistic approaches to trauma-related disease.

##  Key References

- Xu H-K, Liu J-X, Zhou Z-K, Zheng C-X, Sui B-D, Yuan Y, et al. Osteoporosis under psychological stress: mechanisms and therapeutics. life Med 2024:3:lnae009. DOI: 10.1093/lifemedi/lnae009.
  - **○ **This review explores how psychological stress influences bone homeostasis through sympathetic regulation and vascular coupling and highlights mesenchymal stem cell-derived extracellular vesicles as a promising therapeutic approach for stress-related osteoporosis.
- Hofbauer LC, Compston JE, Saag KG, Rauner M, Tsourdi E. Glucocorticoid-induced osteoporosis: novel concepts and clinical implications. The Lancet Diabetes & Endocrinology 2025:13:964–79. DOI: 10.1016/S2213-8587(25)00251-7.
  - **○ **This article reviews the mechanisms of glucocorticoid-induced osteoporosis, emphasizing how glucocorticoids impair bone microarchitecture and fracture risk. Goes on to discuss current diagnostic tools and therapeutic strategies for prevention and treatment.
- Florido A, Velasco ER, Monari S, Cano M, Cardoner N, Sandi C, et al. Glucocorticoid-based pharmacotherapies preventing PTSD. Neuropharmacology 2023:224:109344. DOI: 10.1016/j.neuropharm.2022.109344.
  - **○ **This review evaluates glucocorticoid-based interventions for preventing PTSD, summarizing evidence from both clinical and preclinical studies and discussing mechanisms underlying their potential to reduce trauma-related neurobiological and behavioral changes.
- Hiscox LV, Sharp TH, Olff M, Seedat S, Halligan SL. Sex-Based Contributors to and Consequences of Post-traumatic Stress Disorder. Curr Psychiatry Rep 2023:25:233–45. DOI: 10.1007/s11920-023-01421-z.
  - **○ **This mini-review examines biological factors underlying sex differences in PTSD, summarizes recent evidence on genetic, neuroendocrine, and brain-related mechanisms, as well as implications for treatment and comorbidities.
- Ghițan AF, Gheorman V, Ciurea ME, Gheorman V, Dinescu VC, Ciurea AM, et al. Exploring the Prevalence of PTSD in Hand Trauma: A Comprehensive Study. Brain Sci 2023:13:1438. DOI: 10.3390/brainsci13101438.
  - **○ **This case-control study investigates PTSD symptoms in patients with complex hand and forearm injuries then identifies demographic, clinical, and psychosocial factors such as trauma type, postoperative complications, and lack of family support as key contributors to psychological distress.
- Melillo A, Sansone N, Allan J, Gill N, Herrman H, Cano GM, et al. Recovery-oriented and trauma-informed care for people with mental disorders to promote human rights and quality of mental health care: a scoping review. BMC Psychiatry 2025:25:125. DOI: 10.1186/s12888-025-06473-4.
  - **○ **This review analyzes recovery-oriented and trauma-informed care models in mental health, highlighting their shared principles of autonomy, human rights, and non-coercive practices, and emphasizing the need for standardized frameworks to support implementation.

## Data Availability

No datasets were generated or analysed during the current study.
